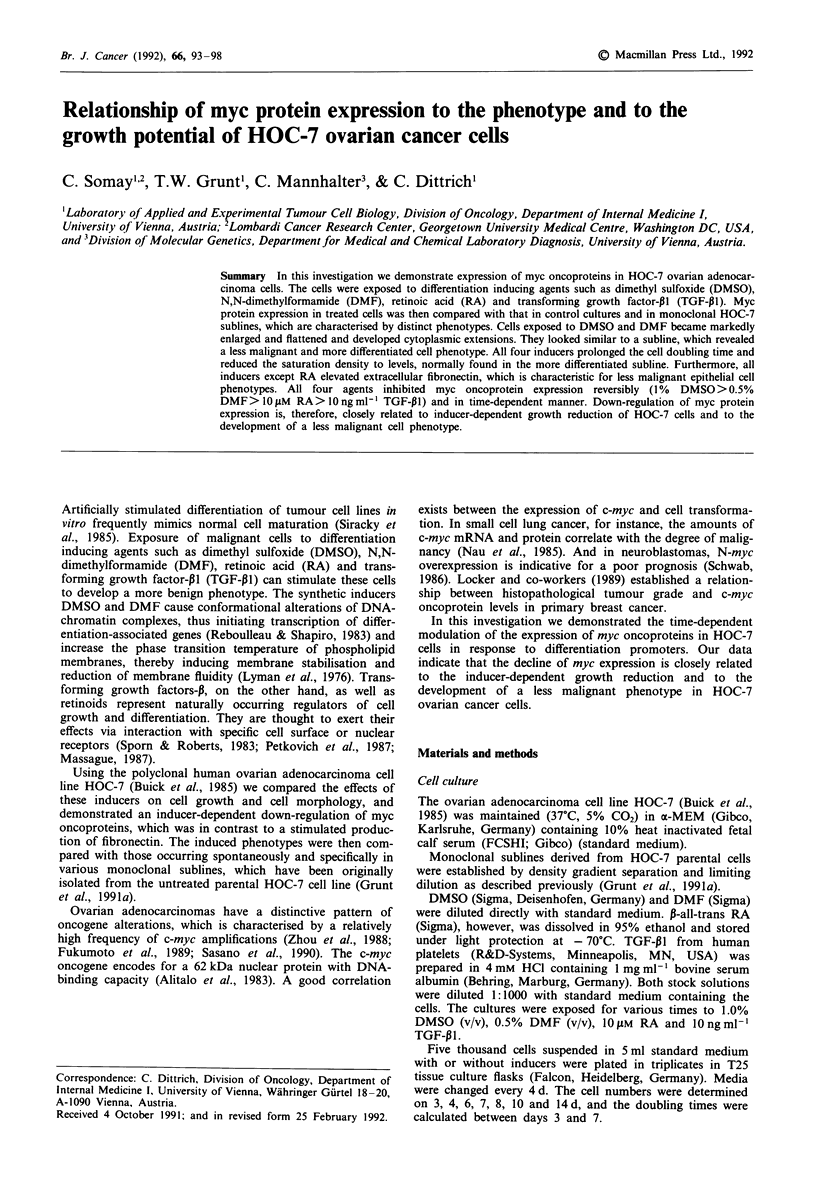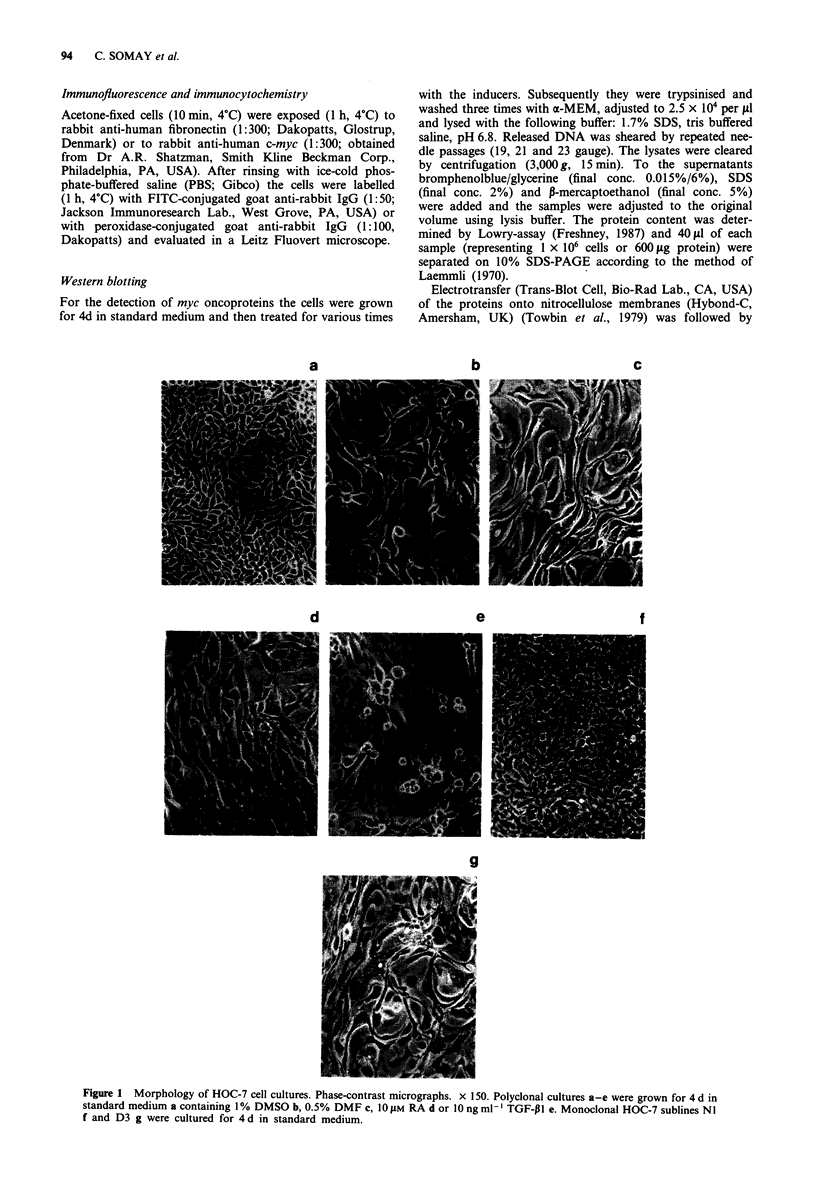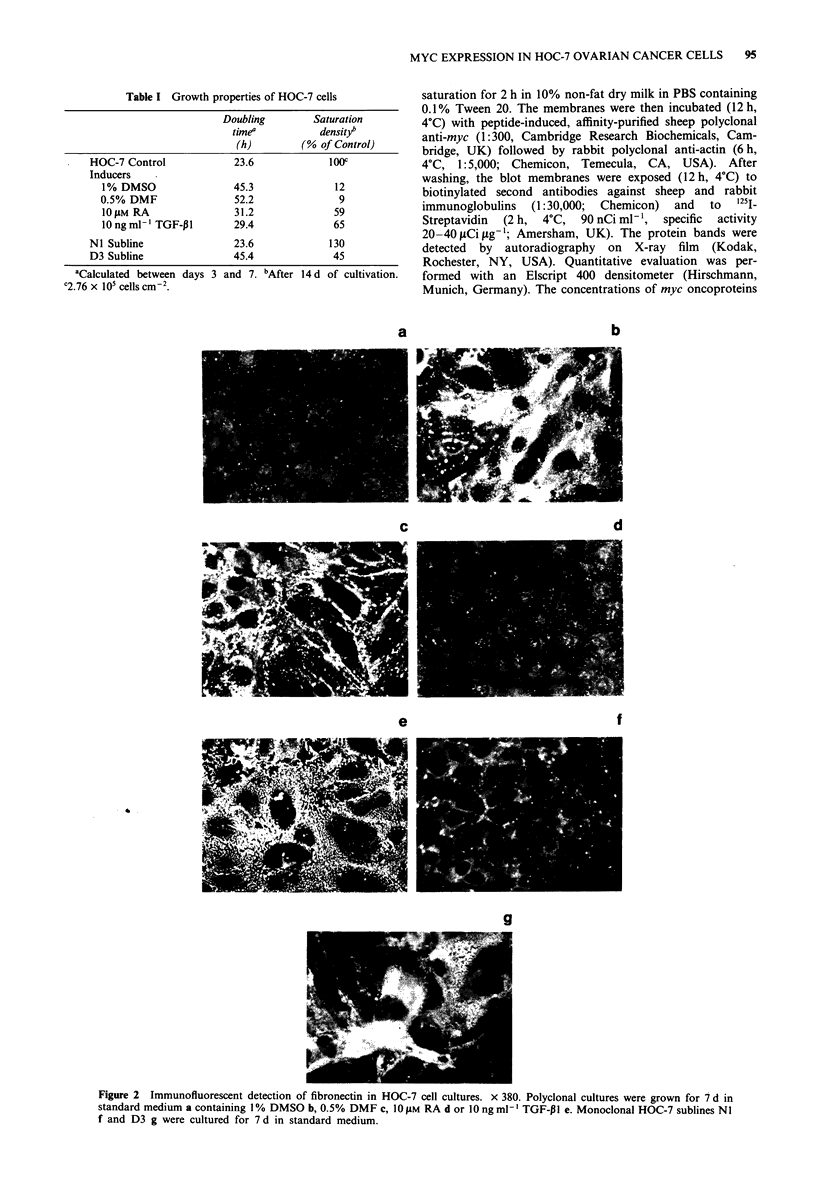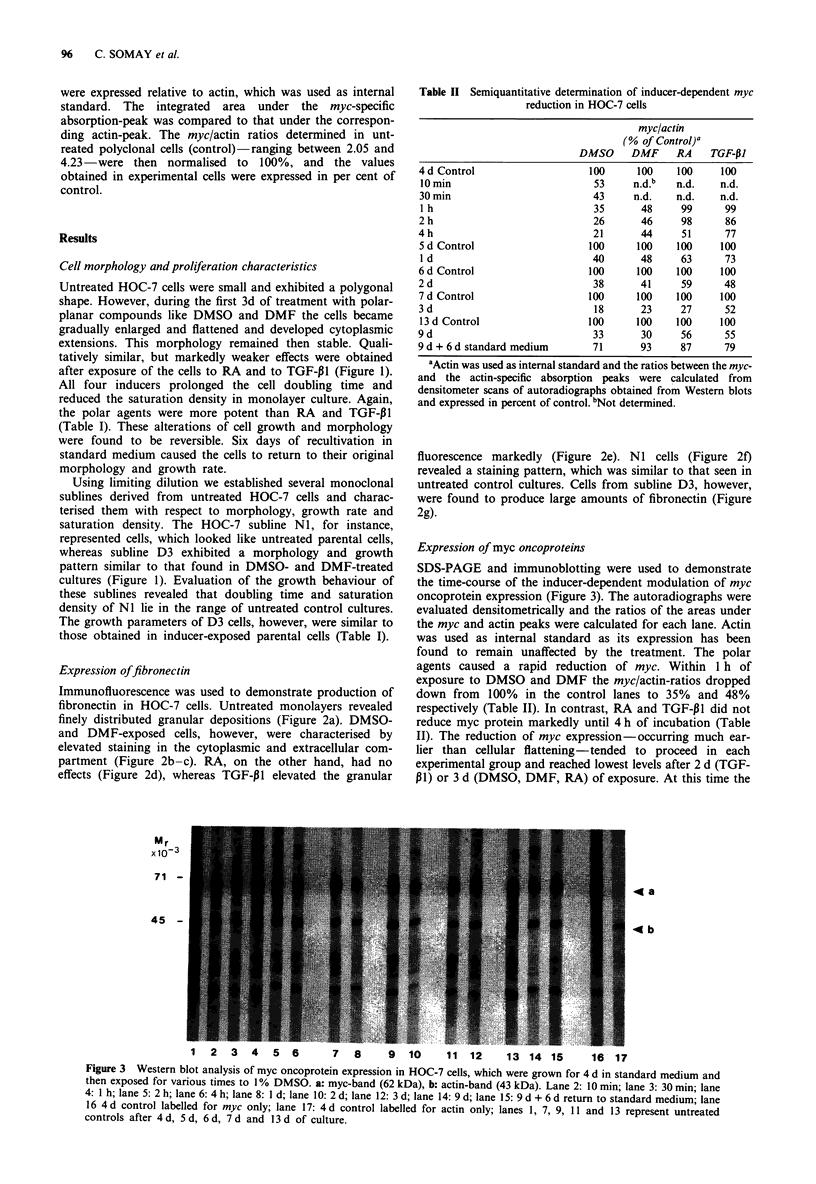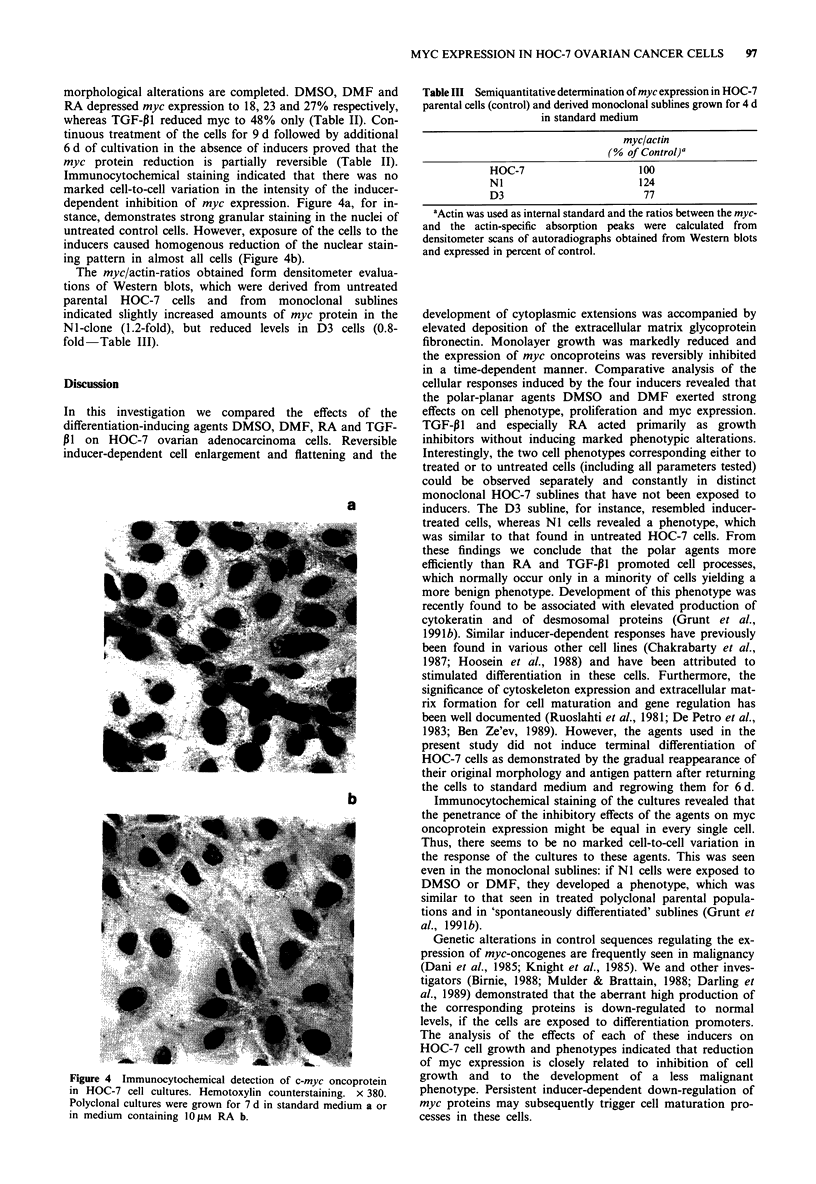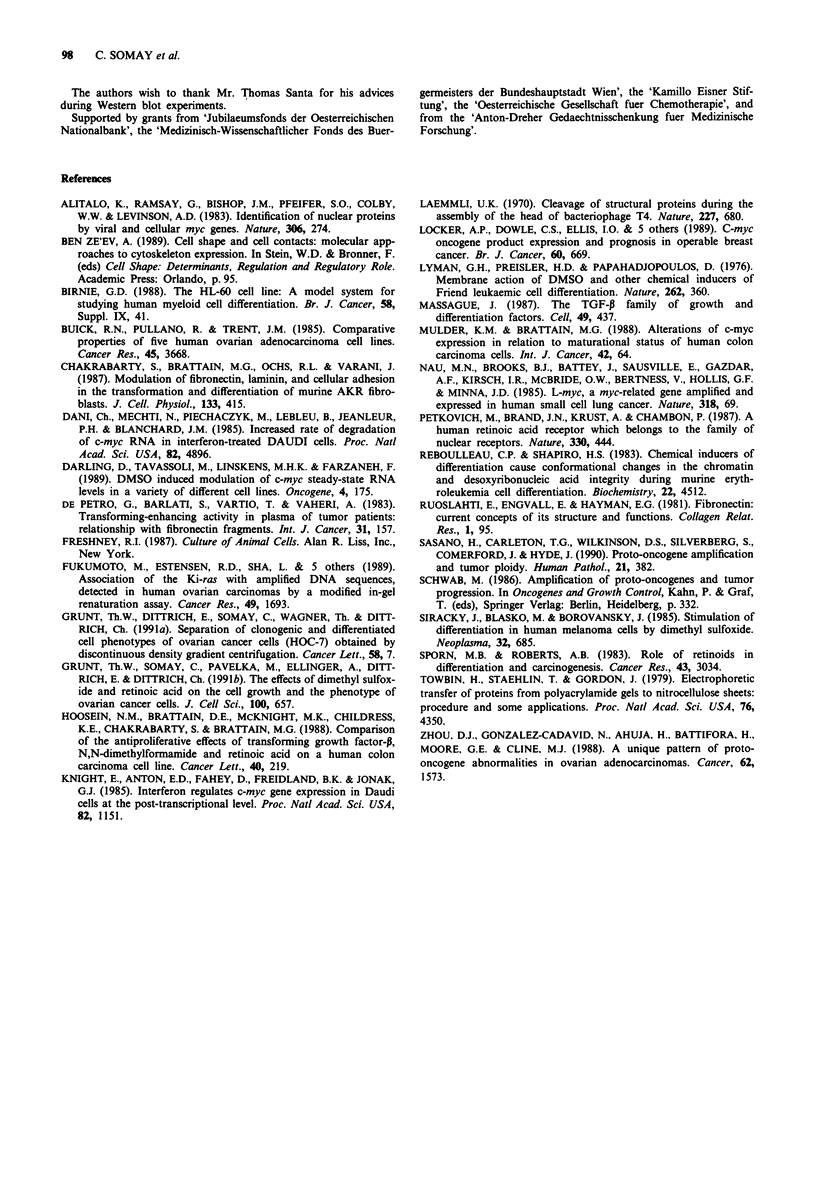# Relationship of myc protein expression to the phenotype and to the growth potential of HOC-7 ovarian cancer cells.

**DOI:** 10.1038/bjc.1992.223

**Published:** 1992-07

**Authors:** C. Somay, T. W. Grunt, C. Mannhalter, C. Dittrich

**Affiliations:** Department of Internal Medicine I, University of Vienna, Austria.

## Abstract

**Images:**


Br. J. Cancer (1992), 66, 93–98 © Macmillan Press Ltd., 1992

### Relationship of myc protein expression to the phenotype and to the growth potential of HOC-7 ovarian cancer cells

C. Somay[1,2], T.W. Grunt[1], C. Mannhalter[3], & C. Dittrich[1]

[1]*Laboratory of Applied and Experimental Tumour Cell Biology, Division of Oncology, Department of Internal Medicine I, University of Vienna, Austria;* [2]*Lombardi Cancer Research Center, Georgetown University Medical Centre, Washington DC, USA, and* [3]*Division of Molecular Genetics, Department for Medical and Chemical Laboratory Diagnosis, University of Vienna, Austria.*

**Summary** In this investigation we demonstrate expression of myc oncoproteins in HOC-7 ovarian adenocarcinoma cells. The cells were exposed to differentiation inducing agents such as dimethyl sulfoxide (DMSO), N,N-dimethylformamide (DMF), retinoic acid (RA) and transforming growth factor-β1 (TGF-β1). Myc protein expression in treated cells was then compared with that in control cultures and in monoclonal HOC-7 sublines, which are characterised by distinct phenotypes. Cells exposed to DMSO and DMF became markedly enlarged and flattened and developed cytoplasmic extensions. They looked similar to a subline, which revealed a less malignant and more differentiated cell phenotype. All four inducers prolonged the cell doubling time and reduced the saturation density to levels, normally found in the more differentiated subline. Furthermore, all inducers except RA elevated extracellular fibronectin, which is characteristic for less malignant epithelial cell phenotypes. All four agents inhibited myc oncoprotein expression reversibly (1% DMSO > 0.5% DMF > 10 μM RA > 10 ng ml$^{-1}$ TGF-β1) and in time-dependent manner. Down-regulation of myc protein expression is, therefore, closely related to inducer-dependent growth reduction of HOC-7 cells and to the development of a less malignant cell phenotype.

Artificially stimulated differentiation of tumour cell lines *in vitro* frequently mimics normal cell maturation (Siracky *et al.*, 1985). Exposure of malignant cells to differentiation inducing agents such as dimethyl sulfoxide (DMSO), N,N-dimethylformamide (DMF), retinoic acid (RA) and transforming growth factor-β1 (TGF-β1) can stimulate these cells to develop a more benign phenotype. The synthetic inducers DMSO and DMF cause conformational alterations of DNA-chromatin complexes, thus initiating transcription of differentiation-associated genes (Reboulleau & Shapiro, 1983) and increase the phase transition temperature of phospholipid membranes, thereby inducing membrane stabilisation and reduction of membrane fluidity (Lyman *et al.*, 1976). Transforming growth factors-β, on the other hand, as well as retinoids represent naturally occurring regulators of cell growth and differentiation. They are thought to exert their effects via interaction with specific cell surface or nuclear receptors (Sporn & Roberts, 1983; Petkovich *et al.*, 1987; Massague, 1987).

Using the polyclonal human ovarian adenocarcinoma cell line HOC-7 (Buick *et al.*, 1985) we compared the effects of these inducers on cell growth and cell morphology, and demonstrated an inducer-dependent down-regulation of myc oncoproteins, which was in contrast to a stimulated production of fibronectin. The induced phenotypes were then compared with those occurring spontaneously and specifically in various monoclonal sublines, which have been originally isolated from the untreated parental HOC-7 cell line (Grunt *et al.*, 1991*a*).

Ovarian adenocarcinomas have a distinctive pattern of oncogene alterations, which is characterised by a relatively high frequency of c-*myc* amplifications (Zhou *et al.*, 1988; Fukumoto *et al.*, 1989; Sasano *et al.*, 1990). The c-*myc* oncogene encodes for a 62 kDa nuclear protein with DNA-binding capacity (Alitalo *et al.*, 1983). A good correlation exists between the expression of c-*myc* and cell transformation. In small cell lung cancer, for instance, the amounts of c-*myc* mRNA and protein correlate with the degree of malignancy (Nau *et al.*, 1985). And in neuroblastomas, N-*myc* overexpression is indicative for a poor prognosis (Schwab, 1986). Locker and co-workers (1989) established a relationship between histopathological tumour grade and c-*myc* oncoprotein levels in primary breast cancer.

In this investigation we demonstrated the time-dependent modulation of the expression of *myc* oncoproteins in HOC-7 cells in response to differentiation promoters. Our data indicate that the decline of *myc* expression is closely related to the inducer-dependent growth reduction and to the development of a less malignant phenotype in HOC-7 ovarian cancer cells.

### Materials and methods

### Cell culture

The ovarian adenocarcinoma cell line HOC-7 (Buick *et al.*, 1985) was maintained (37°C, 5% $CO_2$) in α-MEM (Gibco, Karlsruhe, Germany) containing 10% heat inactivated fetal calf serum (FCSHI; Gibco) (standard medium).

Monoclonal sublines derived from HOC-7 parental cells were established by density gradient separation and limiting dilution as described previously (Grunt *et al.*, 1991*a*).

DMSO (Sigma, Deisenhofen, Germany) and DMF (Sigma) were diluted directly with standard medium. β-all-trans RA (Sigma), however, was dissolved in 95% ethanol and stored under light protection at −70°C. TGF-β1 from human platelets (R&D-Systems, Minneapolis, MN, USA) was prepared in 4 mM HCl containing 1 mg ml$^{-1}$ bovine serum albumin (Behring, Marburg, Germany). Both stock solutions were diluted 1:1000 with standard medium containing the cells. The cultures were exposed for various times to 1.0% DMSO (v/v), 0.5% DMF (v/v), 10 μM RA and 10 ng ml$^{-1}$ TGF-β1.

Five thousand cells suspended in 5 ml standard medium with or without inducers were plated in triplicates in T25 tissue culture flasks (Falcon, Heidelberg, Germany). Media were changed every 4 d. The cell numbers were determined on 3, 4, 6, 7, 8, 10 and 14 d, and the doubling times were calculated between days 3 and 7.

Correspondence: C. Dittrich, Division of Oncology, Department of Internal Medicine I, University of Vienna, Währinger Gürtel 18–20, A-1090 Vienna, Austria.
Received 4 October 1991; and in revised form 25 February 1992.

*Immunofluorescence and immunocytochemistry*

Acetone-fixed cells (10 min, 4°C) were exposed (1 h, 4°C) to rabbit anti-human fibronectin (1:300; Dakopatts, Glostrup, Denmark) or to rabbit anti-human c-*myc* (1:300; obtained from Dr A.R. Shatzman, Smith Kline Beckman Corp., Philadelphia, PA, USA). After rinsing with ice-cold phosphate-buffered saline (PBS; Gibco) the cells were labelled (1 h, 4°C) with FITC-conjugated goat anti-rabbit IgG (1:50; Jackson Immunoresearch Lab., West Grove, PA, USA) or with peroxidase-conjugated goat anti-rabbit IgG (1:100, Dakopatts) and evaluated in a Leitz Fluovert microscope.

*Western blotting*

For the detection of *myc* oncoproteins the cells were grown for 4d in standard medium and then treated for various times with the inducers. Subsequently they were trypsinised and washed three times with α-MEM, adjusted to $2.5 \times 10^4$ per μl and lysed with the following buffer: 1.7% SDS, tris buffered saline, pH 6.8. Released DNA was sheared by repeated needle passages (19, 21 and 23 gauge). The lysates were cleared by centrifugation (3,000 *g*, 15 min). To the supernatants bromphenolblue/glycerine (final conc. 0.015%/6%), SDS (final conc. 2%) and β-mercaptoethanol (final conc. 5%) were added and the samples were adjusted to the original volume using lysis buffer. The protein content was determined by Lowry-assay (Freshney, 1987) and 40 μl of each sample (representing $1 \times 10^6$ cells or 600 μg protein) were separated on 10% SDS-PAGE according to the method of Laemmli (1970).

Electrotransfer (Trans-Blot Cell, Bio-Rad Lab., CA, USA) of the proteins onto nitrocellulose membranes (Hybond-C, Amersham, UK) (Towbin *et al.*, 1979) was followed by

**Figure 1** Morphology of HOC-7 cell cultures. Phase-contrast micrographs. × 150. Polyclonal cultures **a**–**e** were grown for 4 d in standard medium **a** containing 1% DMSO **b**, 0.5% DMF **c**, 10 μM RA **d** or 10 ng ml$^{-1}$ TGF-β1 **e**. Monoclonal HOC-7 sublines N1 **f** and D3 **g** were cultured for 4 d in standard medium.

**Table I** Growth properties of HOC-7 cells

| | *Doubling time*[a] *(h)* | *Saturation density*[b] *(% of Control)* |
|---|---|---|
| HOC-7 Control | 23.6 | 100[c] |
| Inducers | | |
| 1% DMSO | 45.3 | 12 |
| 0.5% DMF | 52.2 | 9 |
| 10 μM RA | 31.2 | 59 |
| 10 ng ml$^{-1}$ TGF-β1 | 29.4 | 65 |
| N1 Subline | 23.6 | 130 |
| D3 Subline | 45.4 | 45 |

[a]Calculated between days 3 and 7. [b]After 14 d of cultivation. [c]2.76 × $10^5$ cells cm$^{-2}$.

saturation for 2 h in 10% non-fat dry milk in PBS containing 0.1% Tween 20. The membranes were then incubated (12 h, 4°C) with peptide-induced, affinity-purified sheep polyclonal anti-*myc* (1:300, Cambridge Research Biochemicals, Cambridge, UK) followed by rabbit polyclonal anti-actin (6 h, 4°C, 1:5,000; Chemicon, Temecula, CA, USA). After washing, the blot membranes were exposed (12 h, 4°C) to biotinylated second antibodies against sheep and rabbit immunoglobulins (1:30,000; Chemicon) and to $^{125}$I-Streptavidin (2 h, 4°C, 90 nCi ml$^{-1}$, specific activity 20–40 μCi μg$^{-1}$; Amersham, UK). The protein bands were detected by autoradiography on X-ray film (Kodak, Rochester, NY, USA). Quantitative evaluation was performed with an Elscript 400 densitometer (Hirschmann, Munich, Germany). The concentrations of *myc* oncoproteins

**Figure 2** Immunofluorescent detection of fibronectin in HOC-7 cell cultures. × 380. Polyclonal cultures were grown for 7 d in standard medium **a** containing 1% DMSO **b**, 0.5% DMF **c**, 10 μM RA **d** or 10 ng ml$^{-1}$ TGF-β1 **e**. Monoclonal HOC-7 sublines N1 **f** and D3 **g** were cultured for 7 d in standard medium.

were expressed relative to actin, which was used as internal standard. The integrated area under the *myc*-specific absorption-peak was compared to that under the corresponding actin-peak. The *myc*/actin ratios determined in untreated polyclonal cells (control)—ranging between 2.05 and 4.23—were then normalised to 100%, and the values obtained in experimental cells were expressed in per cent of control.

### Results

### *Cell morphology and proliferation characteristics*

Untreated HOC-7 cells were small and exhibited a polygonal shape. However, during the first 3d of treatment with polar-planar compounds like DMSO and DMF the cells became gradually enlarged and flattened and developed cytoplasmic extensions. This morphology remained then stable. Qualitatively similar, but markedly weaker effects were obtained after exposure of the cells to RA and to TGF-β1 (Figure 1). All four inducers prolonged the cell doubling time and reduced the saturation density in monolayer culture. Again, the polar agents were more potent than RA and TGF-β1 (Table I). These alterations of cell growth and morphology were found to be reversible. Six days of recultivation in standard medium caused the cells to return to their original morphology and growth rate.

Using limiting dilution we established several monoclonal sublines derived from untreated HOC-7 cells and characterised them with respect to morphology, growth rate and saturation density. The HOC-7 subline N1, for instance, represented cells, which looked like untreated parental cells, whereas subline D3 exhibited a morphology and growth pattern similar to that found in DMSO- and DMF-treated cultures (Figure 1). Evaluation of the growth behaviour of these sublines revealed that doubling time and saturation density of N1 lie in the range of untreated control cultures. The growth parameters of D3 cells, however, were similar to those obtained in inducer-exposed parental cells (Table I).

### *Expression of fibronectin*

Immunofluorescence was used to demonstrate production of fibronectin in HOC-7 cells. Untreated monolayers revealed finely distributed granular depositions (Figure 2a). DMSO- and DMF-exposed cells, however, were characterised by elevated staining in the cytoplasmic and extracellular compartment (Figure 2b–c). RA, on the other hand, had no effects (Figure 2d), whereas TGF-β1 elevated the granular fluorescence markedly (Figure 2e). N1 cells (Figure 2f) revealed a staining pattern, which was similar to that seen in untreated control cultures. Cells from subline D3, however, were found to produce large amounts of fibronectin (Figure 2g).

**Table II** Semiquantitative determination of inducer-dependent *myc* reduction in HOC-7 cells

| | *myc/actin (% of Control)*[a] | | | |
|---|---|---|---|---|
| | *DMSO* | *DMF* | *RA* | *TGF-β1* |
| 4 d Control | 100 | 100 | 100 | 100 |
| 10 min | 53 | n.d.[b] | n.d. | n.d. |
| 30 min | 43 | n.d. | n.d. | n.d. |
| 1 h | 35 | 48 | 99 | 99 |
| 2 h | 26 | 46 | 98 | 86 |
| 4 h | 21 | 44 | 51 | 77 |
| 5 d Control | 100 | 100 | 100 | 100 |
| 1 d | 40 | 48 | 63 | 73 |
| 6 d Control | 100 | 100 | 100 | 100 |
| 2 d | 38 | 41 | 59 | 48 |
| 7 d Control | 100 | 100 | 100 | 100 |
| 3 d | 18 | 23 | 27 | 52 |
| 13 d Control | 100 | 100 | 100 | 100 |
| 9 d | 33 | 30 | 56 | 55 |
| 9 d + 6 d standard medium | 71 | 93 | 87 | 79 |

[a]Actin was used as internal standard and the ratios between the *myc*- and the actin-specific absorption peaks were calculated from densitometer scans of autoradiographs obtained from Western blots and expressed in percent of control. [b]Not determined.

### *Expression of* myc *oncoproteins*

SDS-PAGE and immunoblotting were used to demonstrate the time-course of the inducer-dependent modulation of *myc* oncoprotein expression (Figure 3). The autoradiographs were evaluated densitometrically and the ratios of the areas under the *myc* and actin peaks were calculated for each lane. Actin was used as internal standard as its expression has been found to remain unaffected by the treatment. The polar agents caused a rapid reduction of *myc*. Within 1 h of exposure to DMSO and DMF the *myc*/actin-ratios dropped down from 100% in the control lanes to 35% and 48% respectively (Table II). In contrast, RA and TGF-β1 did not reduce myc protein markedly until 4 h of incubation (Table II). The reduction of *myc* expression—occurring much earlier than cellular flattening—tended to proceed in each experimental group and reached lowest levels after 2 d (TGF-β1) or 3 d (DMSO, DMF, RA) of exposure. At this time the

**Figure 3** Western blot analysis of myc oncoprotein expression in HOC-7 cells, which were grown for 4 d in standard medium and then exposed for various times to 1% DMSO. **a:** myc-band (62 kDa), **b:** actin-band (43 kDa). Lane 2: 10 min; lane 3: 30 min; lane 4: 1 h; lane 5: 2 h; lane 6: 4 h; lane 8: 1 d; lane 10: 2 d; lane 12: 3 d; lane 14: 9 d; lane 15: 9 d + 6 d return to standard medium; lane 16 4 d control labelled for *myc* only; lane 17: 4 d control labelled for actin only; lanes 1, 7, 9, 11 and 13 represent untreated controls after 4 d, 5 d, 6 d, 7 d and 13 d of culture.

morphological alterations are completed. DMSO, DMF and RA depressed *myc* expression to 18, 23 and 27% respectively, whereas TGF-β1 reduced myc to 48% only (Table II). Continuous treatment of the cells for 9 d followed by additional 6 d of cultivation in the absence of inducers proved that the *myc* protein reduction is partially reversible (Table II). Immunocytochemical staining indicated that there was no marked cell-to-cell variation in the intensity of the inducer-dependent inhibition of *myc* expression. Figure 4a, for instance, demonstrates strong granular staining in the nuclei of untreated control cells. However, exposure of the cells to the inducers caused homogenous reduction of the nuclear staining pattern in almost all cells (Figure 4b).

The *myc*/actin-ratios obtained form densitometer evaluations of Western blots, which were derived from untreated parental HOC-7 cells and from monoclonal sublines indicated slightly increased amounts of *myc* protein in the N1-clone (1.2-fold), but reduced levels in D3 cells (0.8-fold—Table III).

### Discussion

In this investigation we compared the effects of the differentiation-inducing agents DMSO, DMF, RA and TGF-β1 on HOC-7 ovarian adenocarcinoma cells. Reversible inducer-dependent cell enlargement and flattening and the development of cytoplasmic extensions was accompanied by elevated deposition of the extracellular matrix glycoprotein fibronectin. Monolayer growth was markedly reduced and the expression of *myc* oncoproteins was reversibly inhibited in a time-dependent manner. Comparative analysis of the cellular responses induced by the four inducers revealed that the polar-planar agents DMSO and DMF exerted strong effects on cell phenotype, proliferation and myc expression. TGF-β1 and especially RA acted primarily as growth inhibitors without inducing marked phenotypic alterations. Interestingly, the two cell phenotypes corresponding either to treated or to untreated cells (including all parameters tested) could be observed separately and constantly in distinct monoclonal HOC-7 sublines that have not been exposed to inducers. The D3 subline, for instance, resembled inducer-treated cells, whereas N1 cells revealed a phenotype, which was similar to that found in untreated HOC-7 cells. From these findings we conclude that the polar agents more efficiently than RA and TGF-β1 promoted cell processes, which normally occur only in a minority of cells yielding a more benign phenotype. Development of this phenotype was recently found to be associated with elevated production of cytokeratin and of desmosomal proteins (Grunt *et al.*, 1991*b*). Similar inducer-dependent responses have previously been found in various other cell lines (Chakrabarty *et al.*, 1987; Hoosein *et al.*, 1988) and have been attributed to stimulated differentiation in these cells. Furthermore, the significance of cytoskeleton expression and extracellular matrix formation for cell maturation and gene regulation has been well documented (Ruoslahti *et al.*, 1981; De Petro *et al.*, 1983; Ben Ze'ev, 1989). However, the agents used in the present study did not induce terminal differentiation of HOC-7 cells as demonstrated by the gradual reappearance of their original morphology and antigen pattern after returning the cells to standard medium and regrowing them for 6 d.

Immunocytochemical staining of the cultures revealed that the penetrance of the inhibitory effects of the agents on myc oncoprotein expression might be equal in every single cell. Thus, there seems to be no marked cell-to-cell variation in the response of the cultures to these agents. This was seen even in the monoclonal sublines: if N1 cells were exposed to DMSO or DMF, they developed a phenotype, which was similar to that seen in treated polyclonal parental populations and in 'spontaneously differentiated' sublines (Grunt *et al.*, 1991*b*).

Genetic alterations in control sequences regulating the expression of *myc*-oncogenes are frequently seen in malignancy (Dani *et al.*, 1985; Knight *et al.*, 1985). We and other investigators (Birnie, 1988; Mulder & Brattain, 1988; Darling *et al.*, 1989) demonstrated that the aberrant high production of the corresponding proteins is down-regulated to normal levels, if the cells are exposed to differentiation promoters. The analysis of the effects of each of these inducers on HOC-7 cell growth and phenotypes indicated that reduction of myc expression is closely related to inhibition of cell growth and to the development of a less malignant phenotype. Persistent inducer-dependent down-regulation of *myc* proteins may subsequently trigger cell maturation processes in these cells.

**Table III** Semiquantitative determination of *myc* expression in HOC-7 parental cells (control) and derived monoclonal sublines grown for 4 d in standard medium

| | *myc/actin* (*% of Control*)[a] |
|---|---|
| HOC-7 | 100 |
| N1 | 124 |
| D3 | 77 |

[a]Actin was used as internal standard and the ratios between the *myc*- and the actin-specific absorption peaks were calculated from densitometer scans of autoradiographs obtained from Western blots and expressed in percent of control.

**Figure 4** Immunocytochemical detection of c-*myc* oncoprotein in HOC-7 cell cultures. Hemotoxylin counterstaining. × 380. Polyclonal cultures were grown for 7 d in standard medium **a** or in medium containing 10 μM RA **b**.

The authors wish to thank Mr. Thomas Santa for his advices during Western blot experiments.

Supported by grants from 'Jubilaeumsfonds der Oesterreichischen Nationalbank', the 'Medizinisch-Wissenschaftlicher Fonds des Buergermeisters der Bundeshauptstadt Wien', the 'Kamillo Eisner Stiftung', the 'Oesterreichische Gesellschaft fuer Chemotherapie', and from the 'Anton-Dreher Gedaechtnisschenkung fuer Medizinische Forschung'.

### References

ALITALO, K., RAMSAY, G., BISHOP, J.M., PFEIFER, S.O., COLBY, W.W. & LEVINSON, A.D. (1983). Identification of nuclear proteins by viral and cellular *myc* genes. *Nature*, **306**, 274.

BEN ZE'EV, A. (1989). Cell shape and cell contacts: molecular approaches to cytoskeleton expression. In Stein, W.D. & Bronner, F. (eds) *Cell Shape: Determinants, Regulation and Regulatory Role*. Academic Press: Orlando, p. 95.

BIRNIE, G.D. (1988). The HL-60 cell line: A model system for studying human myeloid cell differentiation. *Br. J. Cancer*, **58**, Suppl. IX, 41.

BUICK, R.N., PULLANO, R. & TRENT, J.M. (1985). Comparative properties of five human ovarian adenocarcinoma cell lines. *Cancer Res.*, **45**, 3668.

CHAKRABARTY, S., BRATTAIN, M.G., OCHS, R.L. & VARANI, J. (1987). Modulation of fibronectin, laminin, and cellular adhesion in the transformation and differentiation of murine AKR fibroblasts. *J. Cell. Physiol.*, **133**, 415.

DANI, Ch., MECHTI, N., PIECHACZYK, M., LEBLEU, B., JEANLEUR, P.H. & BLANCHARD, J.M. (1985). Increased rate of degradation of c-*myc* RNA in interferon-treated DAUDI cells. *Proc. Natl Acad. Sci. USA*, **82**, 4896.

DARLING, D., TAVASSOLI, M., LINSKENS, M.H.K. & FARZANEH, F. (1989). DMSO induced modulation of c-*myc* steady-state RNA levels in a variety of different cell lines. *Oncogene*, **4**, 175.

DE PETRO, G., BARLATI, S., VARTIO, T. & VAHERI, A. (1983). Transforming-enhancing activity in plasma of tumor patients: relationship with fibronectin fragments. *Int. J. Cancer*, **31**, 157.

FRESHNEY, R.I. (1987). *Culture of Animal Cells*. Alan R. Liss, Inc., New York.

FUKUMOTO, M., ESTENSEN, R.D., SHA, L. & 5 others (1989). Association of the Ki-*ras* with amplified DNA sequences, detected in human ovarian carcinomas by a modified in-gel renaturation assay. *Cancer Res.*, **49**, 1693.

GRUNT, Th.W., DITTRICH, E., SOMAY, C., WAGNER, Th. & DITTRICH, Ch. (1991*a*). Separation of clonogenic and differentiated cell phenotypes of ovarian cancer cells (HOC-7) obtained by discontinuous density gradient centrifugation. *Cancer Lett.*, **58**, 7.

GRUNT, Th.W., SOMAY, C., PAVELKA, M., ELLINGER, A., DITTRICH, E. & DITTRICH, Ch. (1991*b*). The effects of dimethyl sulfoxide and retinoic acid on the cell growth and the phenotype of ovarian cancer cells. *J. Cell Sci.*, **100**, 657.

HOOSEIN, N.M., BRATTAIN, D.E., MCKNIGHT, M.K., CHILDRESS, K.E., CHAKRABARTY, S. & BRATTAIN, M.G. (1988). Comparison of the antiproliferative effects of transforming growth factor-$\beta$, N,N-dimethylformamide and retinoic acid on a human colon carcinoma cell line. *Cancer Lett.*, **40**, 219.

KNIGHT, E., ANTON, E.D., FAHEY, D., FREIDLAND, B.K. & JONAK, G.J. (1985). Interferon regulates c-*myc* gene expression in Daudi cells at the post-transcriptional level. *Proc. Natl Acad. Sci. USA*, **82**, 1151.

LAEMMLI, U.K. (1970). Cleavage of structural proteins during the assembly of the head of bacteriophage T4. *Nature*, **227**, 680.

LOCKER, A.P., DOWLE, C.S., ELLIS, I.O. & 5 others (1989). C-*myc* oncogene product expression and prognosis in operable breast cancer. *Br. J. Cancer*, **60**, 669.

LYMAN, G.H., PREISLER, H.D. & PAPAHADJOPOULOS, D. (1976). Membrane action of DMSO and other chemical inducers of Friend leukaemic cell differentiation. *Nature*, **262**, 360.

MASSAGUE, J. (1987). The TGF-$\beta$ family of growth and differentiation factors. *Cell*, **49**, 437.

MULDER, K.M. & BRATTAIN, M.G. (1988). Alterations of c-myc expression in relation to maturational status of human colon carcinoma cells. *Int. J. Cancer*, **42**, 64.

NAU, M.N., BROOKS, B.J., BATTEY, J., SAUSVILLE, E., GAZDAR, A.F., KIRSCH, I.R., MCBRIDE, O.W., BERTNESS, V., HOLLIS, G.F. & MINNA, J.D. (1985). L-*myc*, a *myc*-related gene amplified and expressed in human small cell lung cancer. *Nature*, **318**, 69.

PETKOVICH, M., BRAND, J.N., KRUST, A. & CHAMBON, P. (1987). A human retinoic acid receptor which belongs to the family of nuclear receptors. *Nature*, **330**, 444.

REBOULLEAU, C.P. & SHAPIRO, H.S. (1983). Chemical inducers of differentiation cause conformational changes in the chromatin and desoxyribonucleic acid integrity during murine erythroleukemia cell differentiation. *Biochemistry*, **22**, 4512.

RUOSLAHTI, E., ENGVALL, E. & HAYMAN, E.G. (1981). Fibronectin: current concepts of its structure and functions. *Collagen Relat. Res.*, **1**, 95.

SASANO, H., CARLETON, T.G., WILKINSON, D.S., SILVERBERG, S., COMERFORD, J. & HYDE, J. (1990). Proto-oncogene amplification and tumor ploidy. *Human Pathol.*, **21**, 382.

SCHWAB, M. (1986). Amplification of proto-oncogenes and tumor progression. In *Oncogenes and Growth Control*, Kahn, P. & Graf, T. (eds), Springer Verlag: Berlin, Heidelberg, p. 332.

SIRACKY, J., BLASKO, M. & BOROVANSKY, J. (1985). Stimulation of differentiation in human melanoma cells by dimethyl sulfoxide. *Neoplasma*, **32**, 685.

SPORN, M.B. & ROBERTS, A.B. (1983). Role of retinoids in differentiation and carcinogenesis. *Cancer Res.*, **43**, 3034.

TOWBIN, H., STAEHLIN, T. & GORDON, J. (1979). Electrophoretic transfer of proteins from polyacrylamide gels to nitrocellulose sheets: procedure and some applications. *Proc. Natl Acad. Sci. USA*, **76**, 4350.

ZHOU, D.J., GONZALEZ-CADAVID, N., AHUJA, H., BATTIFORA, H., MOORE, G.E. & CLINE, M.J. (1988). A unique pattern of proto-oncogene abnormalities in ovarian adenocarcinomas. *Cancer*, **62**, 1573.